# Injectable thermosensitive hydrogel loading erythropoietin and FK506 alleviates gingival inflammation and promotes periodontal tissue regeneration

**DOI:** 10.3389/fbioe.2023.1323554

**Published:** 2024-01-04

**Authors:** Zhongyi Gu, Caiqing Qiu, Ling Chen, Xiaoli Wang

**Affiliations:** ^1^ Department of Periodontology, The Affiliated Yantai Stomatological Hospital, Binzhou Medical University, Yantai, Shandong, China; ^2^ Department of Yantai University Branch, The Affiliated Yantai Stomatological Hospital, Binzhou Medical University, Yantai, Shandong, China

**Keywords:** FK506, hydrogel, periodontitis, periodontium regeneration, erythropoietin

## Abstract

**Background:** Periodontitis is a chronic multifactorial inflammatory disease associated with dysbiotic plaque biofilms and characterized by progressive destruction of the tooth-supporting apparatus. Therefore, there is significant potential in the discovery of drugs that inhibit periodontal inflammatory responses and promote periodontal regeneration.

**Methods:** In this study, we generated a periodontitis rat model to detect the effects of chitosan/β-sodium glycerophosphate (β-GP)/glycolic acid (GA) hydrogel carried Erythropoietin and FK506 (EPO-FK506-CS/β-GP/GA). A total of forty-eight male Wistar rats were used to establish the periodontitis model. Drug injection was administered every 3 days for a total of five times over a 2-week period. After a period of 2 weeks following implantation, the rats underwent anesthesia, and a section of their maxillae encompassing the maxillary first and second molars, along with the alveolar bone, was obtained. micro-CT scanning, histopathology, immunohistochemistry and reverse transcription-quantitative PCR (RT-qPCR) assays were performed. Meanwhile, ELISA assay was performed to detect the levels of inflammatory mediators (TNF-α, IL-6 and IL-1β).

**Results:** The synthesis and characterization of EPO-FK506-CS/β-GP/GA revealed that the hydrogel has stability and sustained release of drugs. The application of FK506+EPO was found to significantly enhance new bone formation in the defect area, as evidenced by the results of HE staining. Additionally, the use of FK506+EPO in the treated groups led to a notable increase in the density of alveolar bone, as observed through micro-CT analysis, when compared to the Model group. EPO-FK506-CS/β-GP/GA hydrogel exhibited notable efficacy in modulating inflammatory mediators (TNF-α, IL-6 and IL-1β). Furthermore, the osteoinductive properties of the EPO-FK506-CS/β-GP/GA hydrogel were extensive, as evidenced by a significant upregulation in the expression of key markers (Collagen I, Runx2, OPN, and OCN) associated with osteoblastic differentiation.

**Conclusion:** Taken together, EPO-FK506-CS/β-GP/GA hydrogel alleviates gingival inflammation and promotes periodontal tissue regeneration in the periodontitis.

## Introduction

Periodontitis is a chronic multifactorial inflammatory disease associated with dysbiotic plaque biofilms and characterized by progressive destruction of the tooth-supporting apparatus, including the gingiva, periodontal ligament, cementum, and alveolar bone (Curtis et al., 2000; Kinane et al., 2017; Lamont et al., 2018). The initiation and propagation of periodontal disease is caused by a dysbiosis of the commensal oral microbiota, which then interacts with the immune system, causing inflammation. (Pirih et al., 2000). As such, the primary objective of periodontitis treatment is to reduce or eliminate periodontal bacterial pathogens. The challenge of administering antibiotics orally to target the infected site in periodontitis results in inadequate drug concentration within the periodontal pocket (Li et al., 2020a). Consequently, there is a pressing need for improved therapeutic interventions to address periodontitis and its related pathologies.

Erythropoietin (EPO) is a biologically active glycoprotein synthesized by the kidneys, with a molecular weight of 30.4 kDa (Deshet-Unger et al., 2020). Its primary functions include the regulation of erythropoiesis and modulation of the immune system (Manto et al., 2022). EPO accounts for approximately 50% of erythropoiesis and exerts its effects by binding to and activating the epigenetic receptor EpoR (Purroy et al., 2017). Additionally, EPO serves as a cytokine with diverse functions, including antioxidant activity and inhibition of apoptosis. In recent years, EPO has gained recognition as a regenerative agent capable of repairing both acute and chronic tissue damage (Guglielmo et al., 2021). The findings of the previous study indicate that EPO enhances and expedites wound healing in mice with heat damage by augmenting vascular proliferation, extracellular matrix maturation, and angiogenesis (Nakamura et al., 2017). Recent research has demonstrated that EPO stimulates the proliferation of vascular structures in damaged nerves, thereby accelerating their recuperation (Zhang et al., 2017). Moreover, the topical administration of erythropoietin (EPO) has been observed to effectively promote the healing of palatal wounds during the third and fourth weeks following a free gingival graft procedure (Sundem et al., 2016; Shao et al., 2022). The administration of a chitosan hydrogel containing aspirin and erythropoietin has been observed to stimulate periodontal regeneration in rats (Xu et al., 2019). Erythropoietin, in addition to its multidirectional differentiation properties, also regulates anti-inflammatory effects, which may facilitate periodontal regeneration.

FK506, a macrolide drug also known as tacrolimus, exhibits potent immunosuppressive properties (Annett et al., 2020). In the context of liver, heart, and kidney transplantation, FK506 effectively suppresses the immune system, thereby preventing organ rejection post-transplantation (Annett et al., 2020; Borges et al., 2023). Additionally, FK506 inhibits the calcineurin enzyme, which in turn suppresses the production of cytokines, including interleukin 2, and ultimately leads to a reduction in inflammation (Akintade and Chaudhuri, 2023). Histological and radiographic studies have demonstrated that FK506 significantly reduces alveolar bone regeneration and granulocyte infiltration caused by periodontitis (Moura Penteado et al., 2017; Murray et al., 2021). The prevention of cytokine expression and minimization of disease progression are mechanisms by which FK506 prevents periodontitis. Prior research has suggested significant antiapoptotic effects of combined EPO plus FK506 on renal tubules, as well as an increase in FK506 blood levels with EPO administration (Jefferies et al., 2022). However, there is a lack of research on the relative effects of this combination in the context of periodontitis.

Currently, a plethora of nanostructured drug delivery systems exist for the treatment of periodontitis, including micelles, liposomes, inorganic nanoparticles, and polymeric nanoparticles (Shen et al., 2020). The biocompatibility, biodegradability, and muco-adhesiveness of chitosan have made it a popular choice for tissue engineering and pharmaceutical applications (Song et al., 2021). When combined with β-sodium glycerophosphate (β-GP), chitosan can form a hydrogel at physiological temperature (Deng et al., 2017; Li et al., 2020b). According to the results of prior research, chitosan hydrogel (CS) has been recognized as a reliable substance for the controlled release of drugs, enabling the precise administration of active pharmaceuticals such as doxycycline, aspirin, and antibiotics to the specific location of the ailment (Zhao et al., 2023). Additionally, CS has been identified as a suitable injectable carrier for the administration of cells and exosomes for the treatment of various ailments. In light of these observations, our study sought to evaluate the efficacy of CS in loading erythropoietin (EPO) and FK506 for the management of periodontitis.

## Materials and methods

### Preparation and characterization of CS/β-GP/GA thermosensitive hydrogel

The CS (type: high molecular weight, molecular weight: 310000–375000 Da, and Sigma, catalog number: 419419) solution was prepared by dissolving 2 g of chitosan into 100 mL of a 2% (v/v) hydrochloric acid (HCl) solution and stirring overnight. In preparation of GA and β-sodium glycerophosphate disodium salt hydrate (β-GP, Mw 216.04, Merck, Darmstadt, Germany) solutions, GA was used at 0.5% (w/v) as well as β-GP at 56% (w/v). A 220 nm filter was utilized for filtering the solutions into the final products.

In a succinct manner, a 5 mL volume of CS was introduced into a 20 mL sterile ampule, subsequently accompanied by the addition of 1 mL of GA and 0.125 mL of β-GP in a gradual manner to the CS solution while maintaining continuous stirring. The pH value was subsequently regulated by employing a 0.1 M NaOH solution. Once the solution attained homogenous mixing, the ampoule was immersed in a water bath at a temperature of 37°C. The ampoule underwent regular examination at 15-s intervals by being extracted from the bath and tilted at a 45-degree angle to determine any changes in the liquid level of the mixed solution. In cases where the liquid level remained unchanged, the ampoule was incubated for an additional 30 s. Following this, the ampoule was completely inverted and observed for any noticeable morphological changes within a 15-s period, indicating the successful formation of the hydrogel. The time taken for the transition from liquid to solid hydrogel formation was defined as the gelling time (GT). These experiments were conducted thrice under identical conditions.

To evaluate the injectability of the hydrogel, a scanning electron microscope (SEM, JSM- 5600 LV, JEOL Ltd., Tokyo, Japan) was employed to assess the surface morphology of the lyophilized CS/β-GP/GA hydrogel.

### Release profile of EPO and FK506 from the hydrogel

To assess the release profile of EPO, a total of 1000 units of EPO and 40 μg FK506 were loaded into a 2 mL thermal-sensitive hydrogel composed of chitosan/β-sodium glycerophosphate/glycolic acid (CS/β-GP/GA) at a pH of 7.0. Subsequently, 2 g of the EPO-FK506-CS/β-GP/GA hydrogel were added to 10 mL of phosphate buffer saline (PBS) and incubated in a water bath at 37°C. At specific time intervals (2 h, 4 h, 8 h, 24 h, 2 days, 4 days, 10 days, and 15 days), the supernatant was aspirated and replaced with 10 mL of fresh PBS. The same experimental procedure was repeated to collect supernatants at the aforementioned time points, which were then utilized for proliferation and differentiation assays. The concentrations of EPO and FK506 in each supernatant were determined using an ELISA assay, and the total amount of drug released was calculated.

### Live/dead cell staining

In order to evaluate the influence of EPO-FK506-CS/β-GP/GA hydrogel on the proliferation of BMSCs, a concentration of 1 × 10^4^ cells/well was cultivated in a 96-well culture plate for a period of 24 h. Following this, BMSCs were introduced into 6-well plates at a density of 2 × 10^5^ cells/well and incubated for 24 h. To evaluate the potential toxicity of the hydrogel, live/dead cell dye (Beyotime, Shanghai, China) was utilized for staining. Live cells were visualized as green due to the excitation of calcein AM, while dead cells were observed as red due to propidium iodide (PI) staining. The stained cells were examined under fluorescence microscopy to detect any adverse effects of the hydrogel.

### Animal model

In order to establish the periodontitis model, a total of forty-eight male Wistar rats with a body weight ranging from 180 to 220 g were utilized. The study was carried out according to the principles of Guide for the Care and Use of Laboratory Animals published by the National Institutes of Health (NIH Publications No. 8023). The animal experiments were approved and conducted in accordance with the Animal Care and Use Committee of The Affiliated Yantai Stomatological Hospital, Binzhou Medical University (NO.2022055).

According to previously studies, the periodontal model was induced by ligatures (Shi et al., 2017; Fan et al., 2023). Briefly, under sterile conditions, a standard ligation wire (0.25 mm) was passed through the proximal and distal adjacent spaces of the bilateral maxillary M1, the wire was then ligated around the dental neck, placed in the gingival sulcus as far as possible, and knotted in the mesiobuccal side of M1. The ligation was monitored every day and re-ligate the molars again if it detached. Periodontal ligation was maintained for 2 weeks.

The rats were divided into six groups (n = 8) in a random manner. These groups included the control group, consisting of healthy rats without periodontitis (referred to as Control), the Model group, which had periodontitis without any treatment, the hydrogel group, where periodontitis was treated with CS/β-GP/GA hydrogels, the FK506 group, where periodontitis was treated with CS/β-GP/GA hydrogels loaded with 3.27 μg/mL FK506, the EPO group, where periodontitis was treated with CS/β-GP/GA hydrogels loaded with 20 U of EPO, and finally, the FK506 + EPO group, where periodontitis was treated with CS/GP/GA hydrogels loaded with both 3.27 μg/mL FK506 and 20 U of EPO. Following this, a volume of 25 μL of PBS was injected into the tissue in the control group, while the other groups received 25 μL of pre-gels (local injection at the mid points of buccal and palatal aspects of maxillary first molars, without removal of ligature). This injection immediately was administered every 3 days after the ligation for a total of five times over a 2-week period (0 days, 3 days, 6 days, 9 days, and 12 days).

Following a 2-week post-implantation period, the rats were subjected to anesthesia, and a segment of their maxillae, comprising the maxillary first and second molars as well as the alveolar bone, was procured and preserved in a 4% PFA solution for a period of 2 days. Subsequently, the specimens were embedded in a 1% agarose medium and subjected to micro-CT scanning using a 19 mm diameter tube.

### Histopathology and immunohistochemistry

Following micro-CT scanning, the samples underwent a 2-month decalcification process in a 10% ethylene diamine tetraacetic acid (EDTA) solution, with weekly refreshment of the EDTA solution. Afterwards, the samples underwent dehydration by employing a graded series of ethanol, followed by embedding in paraffin and sectioning at a thickness of 3.0 μm for the purpose of H&E staining. Subsequently, the microscope was utilized to observe the inflammatory response and histological characteristics of the periodontium.

To assess the inflammatory response, immunohistochemistry (IHC) staining was conducted using anti-rat Collagen Type I (Collagen I, Abcam, Cambridge, Massachusetts, United States of America, ab138492, dilution1:100), Osteocalcin (OCN, Abcam, Cambridge, Massachusetts, United States of America, ab133612, dilution 1:100), Phosphoprotein 1 (OPN, abcam, Cambridge, Massachusetts, United States of America, ab63856, dilution 1:100), and RUNX2 (Abcam, Cambridge, Massachusetts, United States of America, ab192256, dilution 1:100) antibodies, following a standardized protocol outlined in our previous study. Detection of positive staining was achieved using diaminobenzidine tetrachloride (DAB). After that, a microscope was used to quantify the number of positively stained cells located 1 mm above the alveolar crest (between the maxillary first molars and the maxillary second molars) in five fields.

### Reverse transcription-quantitative PCR (RT-qPCR)

Total RNA was extracted from tissues using TRIzol^®^ reagent (Beyotime, Shanghai, China). A SMA2000 spectrophotometer (Thermo Fisher Scientific, Inc.) was used to measure RNA absorbance at 260 nm. Next, the PrimeScript^®^ RT reagent kit (Takara Biotechnology Co., Ltd.) was used for reverse transcription. The sequences of the primers are listed as follows: forward primer for Collagen I: 5’ -GAG​AGA​GCA​TGA​CCG​ATG​GAA​C-3’; reverse primer for Collagen I: 5′-CGT​GCT​GTA​GGT​GAA​TCG​AC-3’; forward primer for OCN: 5′-CAG​ACA​AGT​CCC​ACA​CAG​CA-3’; reverse primer for OCN: 5′-CCA​GCA​GAG​TGA​GCA​GAG​AGA-3’; forward primer for OPN: 5′-ATG​CTA​TCG​ACA​GTC​AGG​CG-3’; reverse primer for OPN 5′-TGC​AGT​GGC​CAT​TTG​CAT​TT-3’; forward primer for RUNX2: 5′-ACT​TCC​TGT​GCT​CGG​TGC​T-3’; reverse primer for RUNX2 5′-GAC​GGT​TAT​GGT​CAA​GGT​GAA-3’; forward primer for GAPDH: 5′-ATG​GCT​ACA​GCA​ACA​GGG​T-3’; reverse primer for GAPDH 5′-TTA​TGG​GGT​CTG​GGA​TGG-3’. Reaction conditions: 95°C: 5 min; 95°C: 15 s; 58°C: 15 s; 72°C: 32 s; 40 cycles. GAPDH served as an internal reference gene. The primer sequences were synthesized by Sangon Biotech Co., Ltd. Target gene expressions were performed using 2^−ΔΔCT^ method.

### Statistical analysis

The experimental data were analyzed using IBM SPSS Statistics (version 19.0, SPSS Inc., Chicago, IL, United States of America). To determine significant differences in the data, a one-way analysis of variance (ANOVA) followed by Tukey’s post-test was conducted. Statistical significance was defined as *p* < 0.05.

## Results

### Synthesis and characterization of CS/β-GP/GA hydrogels loaded with FK506/EPO

**FIGURE 1:**
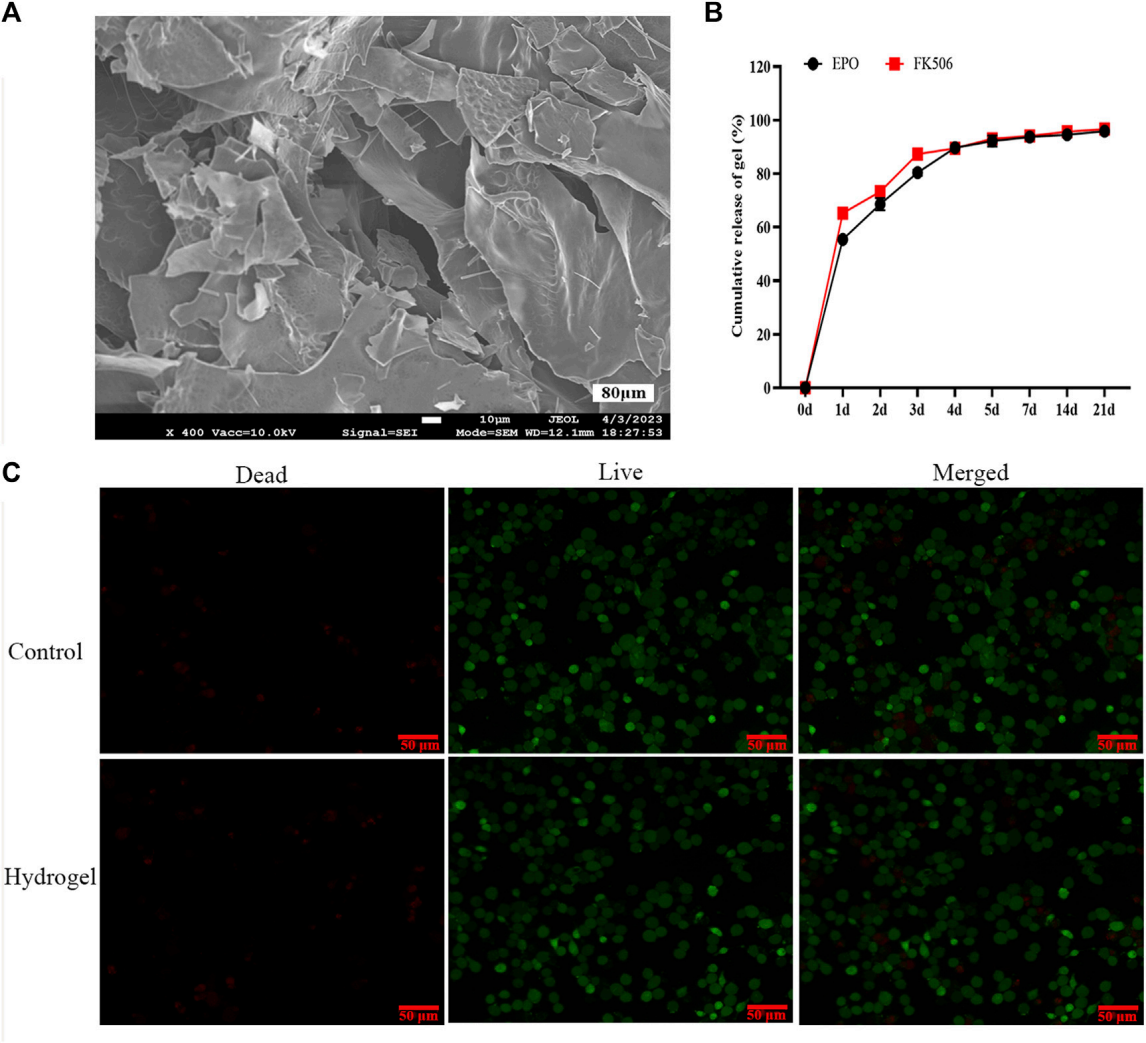
Photographic illustration, characterization and toxicity of the CS/β-GP/GA hydrogels. **(A)** A representative SEM image of the hydrogels; **(B)** The releasing curves of FK506 and EPO; **(C)** *In vitro* evaluation of the toxicity of the hydrogels via live/dead cell staining.

The pre-gels, composed of CS/β-GP/GA, demonstrated a translucent and slightly viscous liquid state at ambient temperature. When the temperature surpassed 37°C, a solgel transition took place, leading to the creation of a hydrogel within a duration of 5 min. Examination of scanning electron microscope (SEM) images (Figure 1A) indicated that the freeze-dried hydrogels exhibited pore sizes spanning from 40 to 80 μm, enabling the sustained release of drugs, cellular penetration, and nutrient/waste exchange. The observed stability of our injectable and thermosensitive hydrogel suggests promising potential for practical applications. According to the data presented in Figure 1B, the release profiles of FK506 and EPO exhibited an initial burst release phenomenon within the initial 3-day period. During this timeframe, the quantities of FK506 and EPO released reached 81.5% and 87.3%, respectively.

### Toxicity of CS/β-GP/GA hydrogels *in vitro*

Live/dead staining results revealed no statistically significant difference in rat bone marrow stromal cells (BMSCs) proliferation between the four experimental groups and the control group (Figure 1C). Moreover, the findings indicated that CS/β-GP/GA hydrogels devoid of pharmaceutical agents did not elicit cytotoxicity or any detrimental impacts in an *in vitro* setting.

### *In vivo* anti-inflammation effect of the CS/β-GP/GA hydrogels loaded with FK506/EPO

**FIGURE 2:**
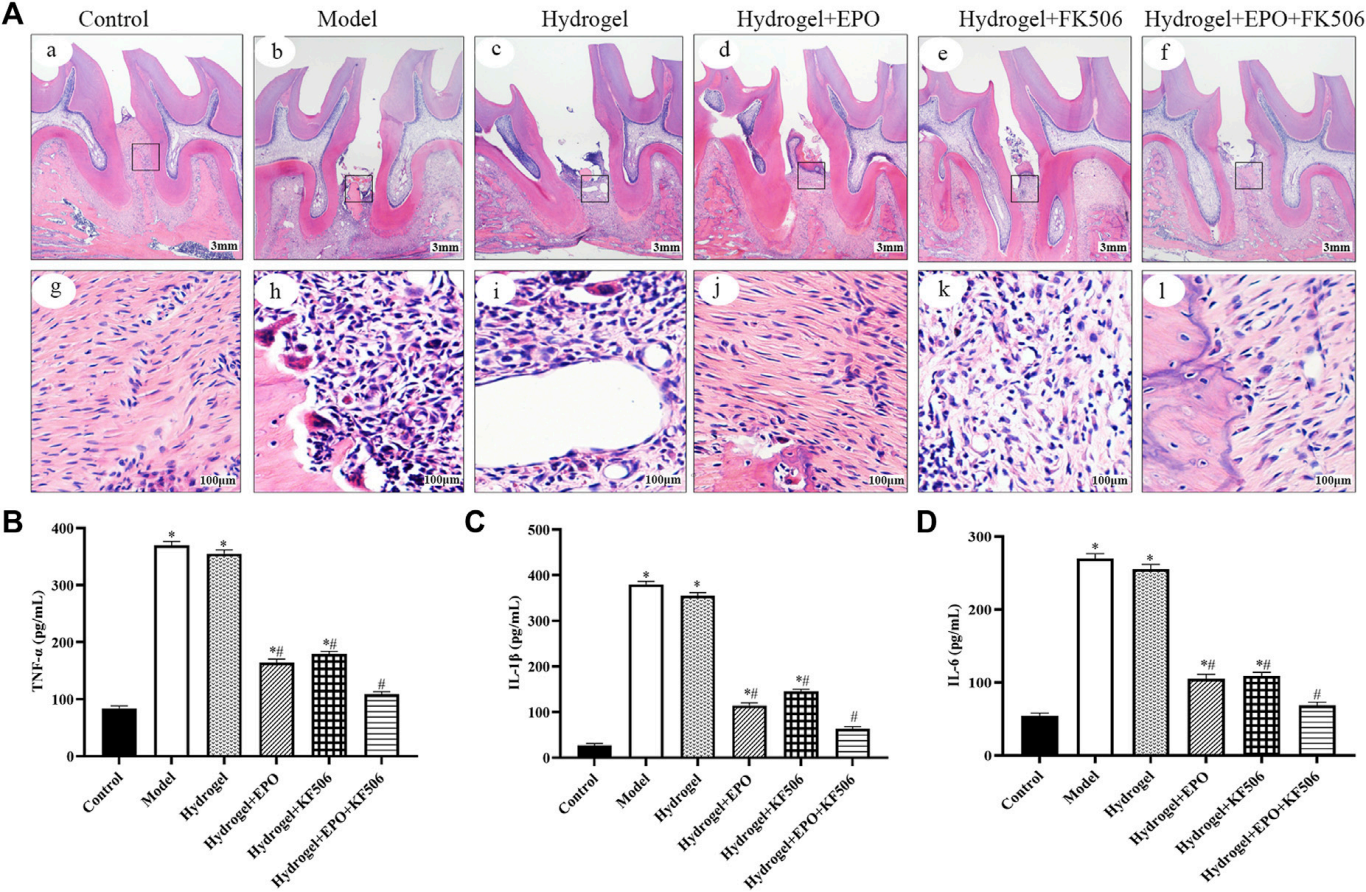
Anti-inflammation and periodontium regeneration effects of the hydrogels in periodontitis rat model. **(A)** Histopathology examination; **(B)** TNF-α levels; **(C)** IL-1β levels; **(D)** IL-6 levels. ^*^ *p* < 0.05 vs. Control group; ^#^ *p* < 0.05 vs. Model group.

The *in vivo* anti-inflammatory effect of CS/β-GP/GA hydrogels loaded with FK506/EPO was investigated to determine their ability to inhibit periodontal inflammation, which is known to hinder new bone formation. The inflammatory reaction was assessed to confirm the inhibitory effect of FK506/EPO released from the hydrogels. As shown in Figures 2B–D, compared to the control group, the Model group exhibited significantly elevated levels of serum TNF-α, IL-1β, and IL-6. However, treatment with FK506/EPO effectively suppressed the increase in serum TNF-α, IL-1β, and IL-6 induced by experimental periodontitis. The group treated with a combination of EPO and FK506 demonstrated superior effects compared to the groups treated with EPO or FK506 alone.

### Periodontium regeneration by CS/β-GP/GA hydrogels loaded with FK506/EPO

As shown in Figure 2A, the histological examination of specimens from the control group revealed the presence of a normal physiological periodontium, characterized by well-defined gingiva, periodontal ligament, cementum, and alveolar bone. In contrast, the ligation group exhibited absorption in the alveolar bone between the first and second molars. Additionally, observations included hyperplasia of gingival epithelial spikes, thickening of the stratum spinosum, and significant degradation of collagen fibers. However, treatment with FK506/EPO appeared to effectively restore tissue integrity in the ligation group.

**FIGURE 3:**
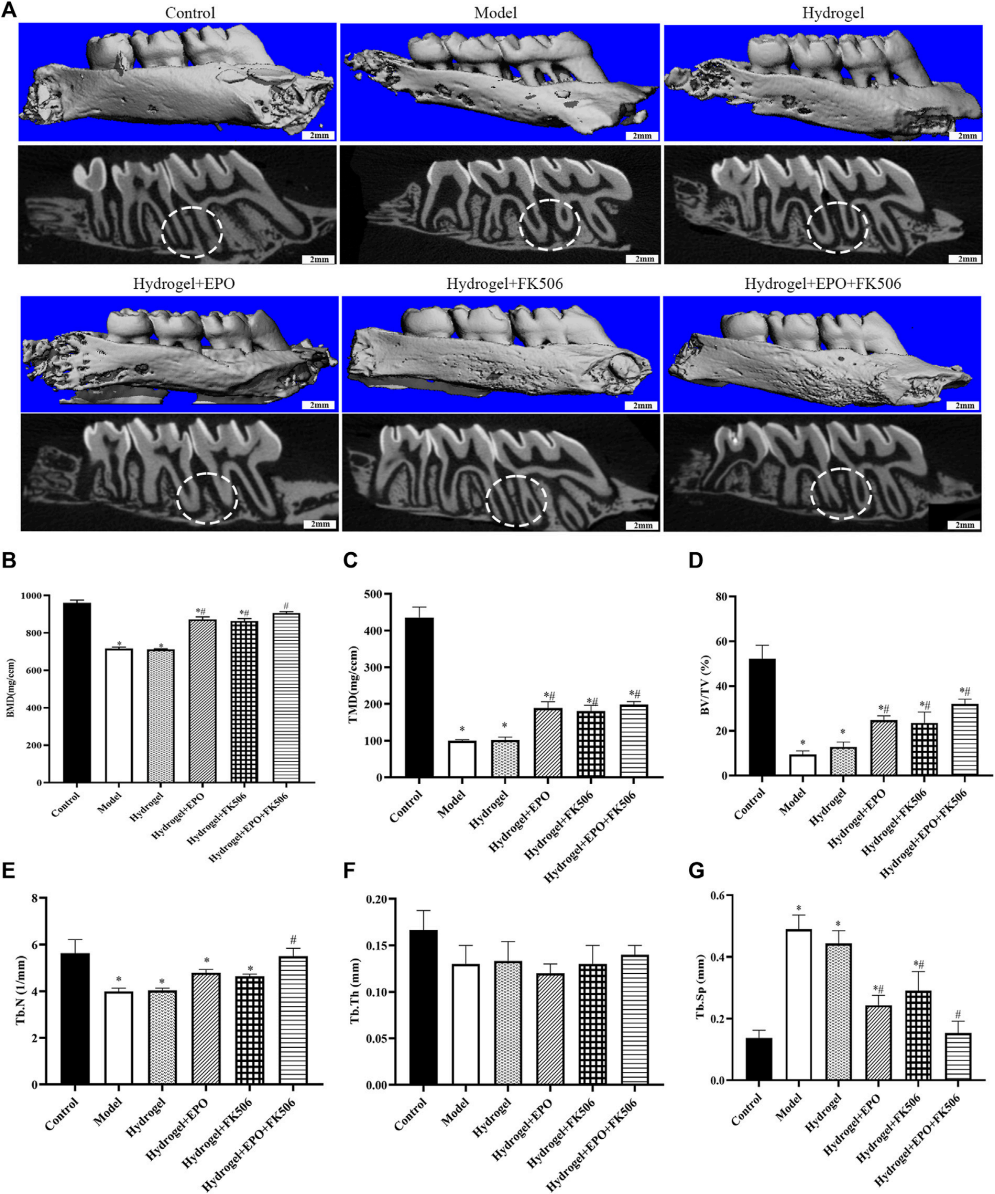
Regeneration of the alveolar bone evaluated by micro-CT. **(A)** Micro-CT images of maxillary alveolar bone surrounding the maxillary first molars (M1) and maxillary second molars (M2) 2 weeks after the treatment; **(B)** bone mineral density (BMD, mg/ccm); **(C)** tissue mineralized density (TMD, mg/ccm); **(D)** bone volume fraction (BV/TV, %); **(E)** trabecular number (Tb. N, 1/mm); **(F)** trabecular thickness (Tb. Th, mm); **(G)** trabecular separation (Tb. Sp, mm). ^*^ *p* < 0.05 vs. Control group; ^#^ *p* < 0.05 vs. Model group.

Figure 3 illustrates the analysis of Micro-CT images and data, which reveals a noteworthy decrease in bone mineral density (BMD, mg/ccm), tissue mineralized density (TMD, mg/ccm), bone volume fraction (BV/TV, %), trabecular thickness (Tb. Th, mm), and trabecular number (Tb. N, 1/mm) in the periodontitis group. The Model group, on the other hand, exhibits an increase in trabecular separation (Tb. Sp, mm) over the Control group. These findings provide further evidence for the successful establishment of the periodontitis model, as they indicate a significant decline in alveolar bone mass and mineralization. Furthermore, when compared to the Model group, the periodontitis group exhibited a notable increase in BMD, TMD, BV/TV, and Tb. N based on Micro-CT images and data. Conversely, there was an observed decrease in Tb. Sp. The group treated with a combination of EPO and FK506 demonstrated superior effects compared to the groups treated with EPO or FK506 alone.

### CS/β-GP/GA hydrogels loaded with FK506/EPO affects bone turnover and metabolism

**FIGURE 4:**
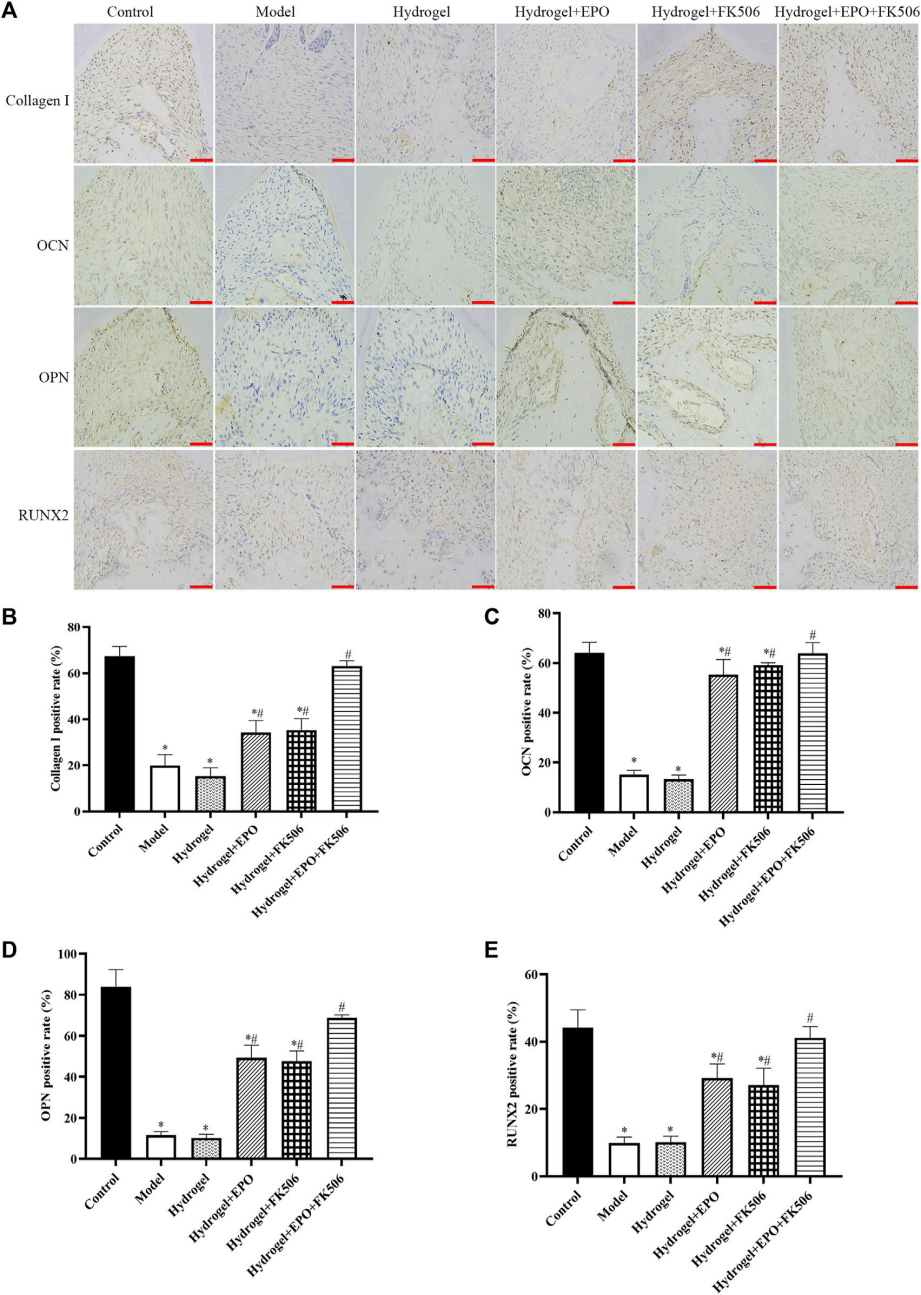
Effects of the hydrogels on osteogenic markers (Collagen I, OCN, OPN, RUNX2) in periodontitis rat model. **(A)** Immunohistochemical images (scar bar = 50 μm); **(B)** Collagen I positive rate; **(C)** OCN positive rate; **(D)** OPN positive rate; **(E)** RUNX2 positive rate. ^*^ *p* < 0.05 vs. Control group; ^#^ *p* < 0.05 vs. Model group.

In order to assess the impact of FK506/EPO on osteogenic differentiation in rats with periodontitis, we utilized immunohistochemistry methods to measure the levels of osteogenic markers, namely, Collagen I, OCN, OPN, and RUNX2. As depicted in Figure 4, the Model group exhibited a noticeable decrease in osteogenic differentiation, as evidenced by the significantly reduced levels of Collagen I (Figure 4B), OCN (Figure 4C), OPN (Figure 4D), and RUNX2 (Figure 4E). Conversely, the administration of FK506/EPO mitigated the suppression of osteogenic differentiation in periodontitis, leading to a significant increase in the levels of osteogenic markers (Figure 4). The group treated with a combination of EPO and FK506 demonstrated superior effects compared to the groups treated with EPO or FK506 alone.

**FIGURE 5:**
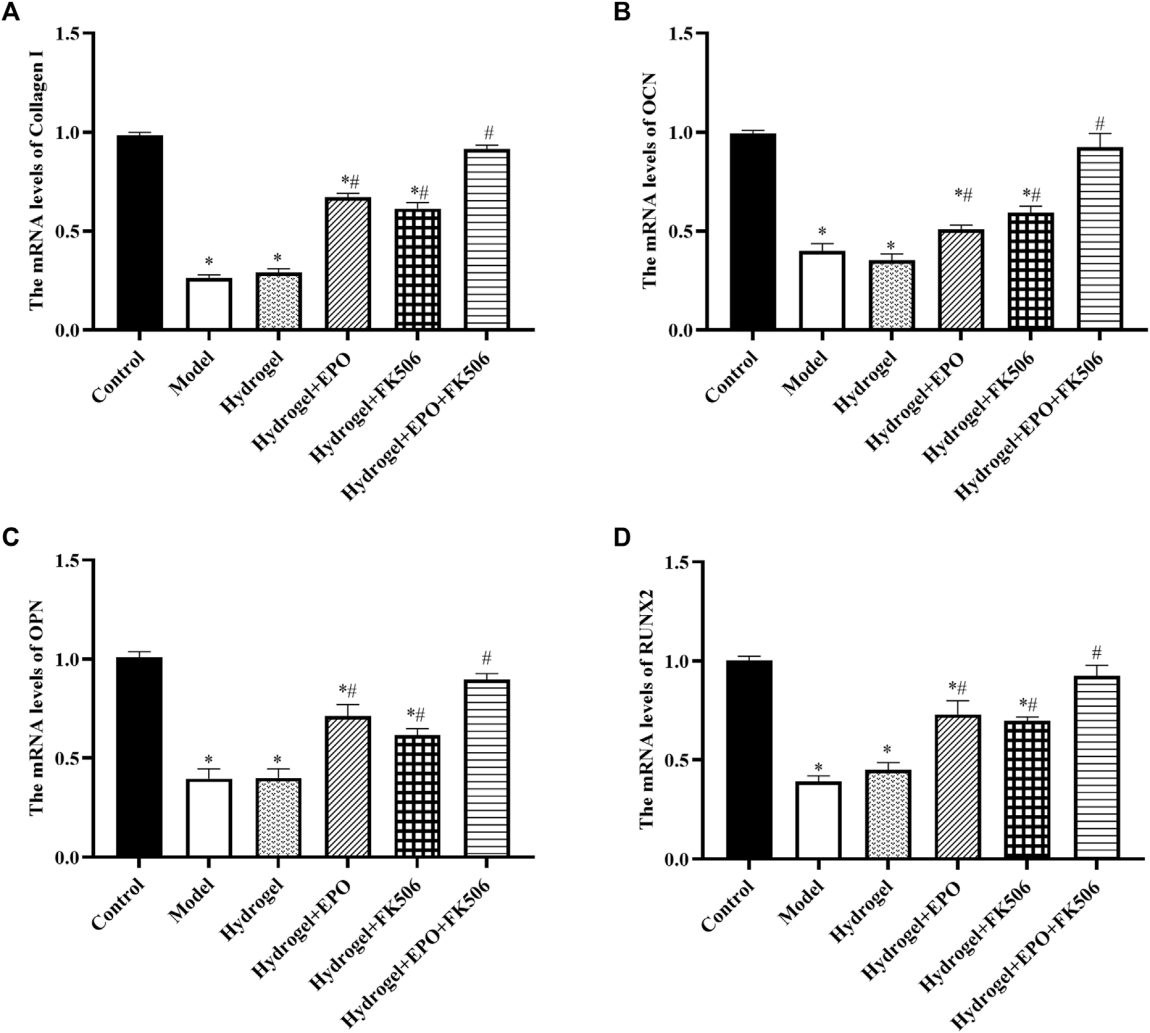
Effects of the hydrogels on mRNA levels of osteogenic markers (Collagen I, OCN, OPN, RUNX2). **(A)** Collagen I expression; **(B)** OCN expression; **(C)** OPN expression; **(D)** RUNX2 expression. ^*^ *p* < 0.05 vs. Control group; ^#^ *p* < 0.05 vs. Model group.

Consistently, the mRNA levels of Collagen I (Figure 5A), OCN (Figure 5B), OPN (Figure 5C), and RUNX2 (Figure 5D) were consistent with the immunohistochemistry results.

## Discussion

Periodontitis is a chronic infectious disease characterized by the destruction of periodontal tissues, typically resulting in inflammation of the gums, formation of peripheral pockets, resorption of alveolar bone, loosening and displacement of teeth, thereby serving as the primary factor contributing to tooth loss among adults. In this present study, we investigated the impact of EPO-FK506-CS/β-GP/GA hydrogel on inflammation and osteogenic differentiation in the context of periodontitis. The findings of the study demonstrated that the EPO-FK506-CS/β-GP/GA hydrogel exhibited notable efficacy in modulating inflammatory mediators, specifically TNF-α, IL-6 and IL-1 β in the periodontal milieu. Furthermore, the osteoinductive properties of the EPO-FK506-CS/β-GP/GA hydrogel were extensive, as evidenced by a significant upregulation in the expression of key markers associated with osteoblastic differentiation, including Collagen I, Runx2, OPN and OCN.

The CS/β-GP/GA hydrogel is considered an optimal material for drug controlled release systems due to its favorable fluidity at room temperature, bacteriostatic properties, biocompatibility, and unique ability to enhance cell osteogenic differentiation (Xu et al., 2019). In comparison to the conventional CS/β-GP hydrogel, CS/β-GP/GA hydrogelis has the advantage of faster gel formation at 37°C, convenient application, injection filling of irregular cavities, and controlled release of active ingredients. The optimization of the CS/β-GP/GA hydrogel system was achieved in this experiment through the incorporation of gelatin as an amphoteric electrolyte. As a result of rapid cross-linking of CS with sodium glycerophosphate between 34°C and 40°C, the gel formed at body temperature rapidly. Clinical applications are made easier due to this characteristic. Moreover, scanning electron microscopy revealed that the EPO-FK506-loaded chitosan hydrogel had a loose and porous structure, which is essential for cell survival.

Based on prior research, it has been observed that erythropoietin (EPO) hinders proinflammatory pathways and apoptosis, promotes vascularization, facilitates endochondral ossification, and enhances osteoblastogenesis (Shao et al., 2022). Earlier investigations have indicated that FK506 demonstrates diverse therapeutic effects, including antifungal, neuroprotective, neuroregenerative, and immunosuppressive properties (Annett et al., 2020). The immunosuppressive effect of FK506 is ascribed to its capacity to impede T-cell proliferation. FK506 binding proteins, which are classified as immunophilins, play vital roles in regulating signaling pathways related to inflammation, adaptive immune responses, cancer, and developmental biology (Zuo et al., 2020; Daeschler et al., 2023). Notably, FK506 exhibits diverse neurological activities, including neuroprotection in stroke models of focal cerebral ischemia and neuroregeneration in animal models of neurodegenerative diseases and nerve injury (Zuo et al., 2021). In the present study, it was observed that the administration of CS/β-GP/GA hydrogels loaded with FK506/EPO resulted in a significantly greater relative height of the residual alveolar ridge compared to the Model group. This finding suggests that the FK506+EPO-treated groups exhibited a higher degree of bone formation than the Model group. Histological analysis using HE staining further revealed a marked increase in new bone formation in the defect area following FK506+EPO treatment. Additionally, micro-CT analysis demonstrated a significant increase in alveolar bone density in the FK506+EPO-treated groups compared to the Model group. Moreover, these results demonstrated that FK506+EPO treatment enhances bone formation.

The role of inflammation in the development of periodontal disease has been established. Inflammatory mediators such as TNF-α, IL-6, and IL-1β play a significant role in influencing the regenerative potential of the tooth within the periodontal environment. Notably, TNF-α, a crucial member of the TNF family, serves as the principal inflammatory factor governing tissue degradation in periodontitis and participates in various inflammatory responses (Otenio et al., 2012; Abidi et al., 2022). Patients with periodontitis exhibit elevated levels of salivary IL-1β, which serves as a significant indicator in gingival crevicular fluid, reflecting both the inactive and active phases of periodontitis (Cheng et al., 2020). Additionally, recent research has linked IL-6 to the progression of periodontal disease and the exacerbation of tissue degradation. Numerous cross-sectional studies have consistently demonstrated that individuals with periodontitis display higher serum IL-1β expression levels compared to healthy individuals, and these levels positively correlate with the clinical parameters of periodontitis (Ghassib et al., 2019). In the current study, the expression levels of inflammatory mediators, including TNF-α, IL-6 and IL-1β were all significantly decreased via treated with CS/β-GP/GA hydrogels loaded with FK506/EPO.

Growth factors are essential in the context of periodontal regeneration due to their ability to enhance the proliferation, differentiation, and tissue-forming capacities of stem cells (Li and Peng, 2020). Molecular components of the osteogenic process supervise and induce osteogenesis, while facilitating intercellular and intracellular communication. The expression levels of osteogenic differentiation markers, including OPN, Collagen I, RUNX family transcription factor, and OCN, exhibited a significant increase in the differentiated cells (Thanakun et al., 2019; Zhang et al., 2019). Exceptional biomaterials not only function as scaffolds but also stimulate the osteogenic differentiation of cells.

RUNX2 is a crucial transcription factor that plays a pivotal role in the initiation and regulation of early osteogenesis (Wang et al., 2023). In addition to its role in osteogenic differentiation and bone formation, it activates downstream factors such as RUNX2, OPN, OCN, and Collagen, among others (Qiu et al., 2021). The expression of this protein remains elevated in immature osteoblasts, but gradually diminishes as an osteoblast matures. The osteoblast manufactures OCN exclusively when the bone matrix is mineralized, and it is a non-collagenous protein (Kuchler et al., 2021). Hence, it serves as an important indicator of osteogenic activity during late maturation. Bone remodeling is accompanied by an increase in the expression of OPN in osteoblasts following an increase in bone matrix mineralization. In addition to facilitating osteoblast adhesion, this protein is also involved in mineralization and adsorption. Additionally, RUNX2 is a crucial transcription factor that initiates osteogenesis. The gene OCN is the subject of regulation by RUNX2, serving as its target gene. The observed elevation in OCN gene expression serves as empirical support for the progression of osteoblast maturation (Cui et al., 2023). Consequently, OCN is widely acknowledged as a marker indicative of the advanced stages of osteogenic differentiation (Hakki et al., 2021). In the current study, by treating CS/β-GP/GA hydrogels loaded with FK506/EPO, osteoblast differentiation markers such as Collagen, Runx2, OPN, and OCN were increased significantly.

In summary, our study suggests that the utilization of the EPO-FK506-CS/β-GP/GA hydrogel system exhibits potential in suppressing periodontal inflammation, facilitating periodontal tissue restoration and regeneration, and holds promising prospects for local drug therapy in periodontitis. Nevertheless, the precise underlying mechanism remains unclear. *In vitro* investigations are being pursued to further elucidate and clarify.

## Conclusion

In conclusion, in the current research, we presented the successful development of a thermosensitive injectable hydrogel that exhibits loose porous water and excellent biocompatibility. Our results demonstrated that EPO-FK506-CS/β-GP/GA hydrogel exhibited notable efficacy in modulating inflammatory mediators, specifically TNF-α, IL-6 and IL-1β. Furthermore, the osteoinductive properties of the EPO-FK506-CS/β-GP/GA hydrogel were extensive, as evidenced by a significant upregulation in the expression of key markers (Collagen I, Runx2, OPN, and OCN) associated with osteoblastic differentiation. EPO-FK506-CS/β-GP/GA hydrogel alleviates gingival inflammation and promotes alveolar bone regeneration in the periodontitis, exhibiting significant research potential and promising prospects for application in the field of periodontal tissue regeneration.

## Data Availability

The raw data supporting the conclusion of this article will be made available by the authors, without undue reservation.
